# P-937. Clinicomicrobiological Risk Factors for Infective Endocarditis in Viridans Group Streptococci Bacteremia

**DOI:** 10.1093/ofid/ofae631.1128

**Published:** 2025-01-29

**Authors:** Jiyeon Bae, Jae Hyeon Park, Minkyeong Lee, Hyeon Jae Jo, Chan Mi Lee, Chang Kyung Kang, Pyoeng Gyun Choe, Wan Beom Park, Nam Joong Kim, Inho Kim, Myoung-don Oh

**Affiliations:** Ewha Womans University Mokdong Hospital, Seoul, Seoul-t'ukpyolsi, Republic of Korea; Seoul National University Hospital, Seoul, Seoul-t'ukpyolsi, Republic of Korea; Chungbuk National University Hospital, Cheongju City, Ch'ungch'ong-bukto, Republic of Korea; Seoul National University College of Medicine, Seoul, Seoul-t'ukpyolsi, Republic of Korea; Seoul National University College of Medicine, Seoul, Seoul-t'ukpyolsi, Republic of Korea; Seoul National University College of Medicine, Seoul, Seoul-t'ukpyolsi, Republic of Korea; Seoul National University College of Medicine, Seoul, Seoul-t'ukpyolsi, Republic of Korea; Seoul National University College of Medicine, Seoul, Seoul-t'ukpyolsi, Republic of Korea; Seoul National University College of Medicine, Seoul, Seoul-t'ukpyolsi, Republic of Korea; Seoul National University Hospital, Seoul, Seoul-t'ukpyolsi, Republic of Korea; Department of Internal Medicine, Seoul National University College of Medicine, Seoul, Korea, Seoul, Seoul-t'ukpyolsi, Republic of Korea

## Abstract

**Background:**

Viridans group streptococci (VGS) persist as the dominant cause of community-acquired native-valve endocarditis, contributing to 20% of cases. The incidence of streptococcal infective endocarditis (IE) exceeds that of *Staphylococcus aureus* IE, notably in Asia. However, the risk factors that guide the decision to conduct echocardiography to rule out IE in patients with VGS bloodstream infections (BSIs) remain unclear. Therefore, our study aimed to identify independent risk factors for IE in patients with VGS BSIs.

**Figure:**
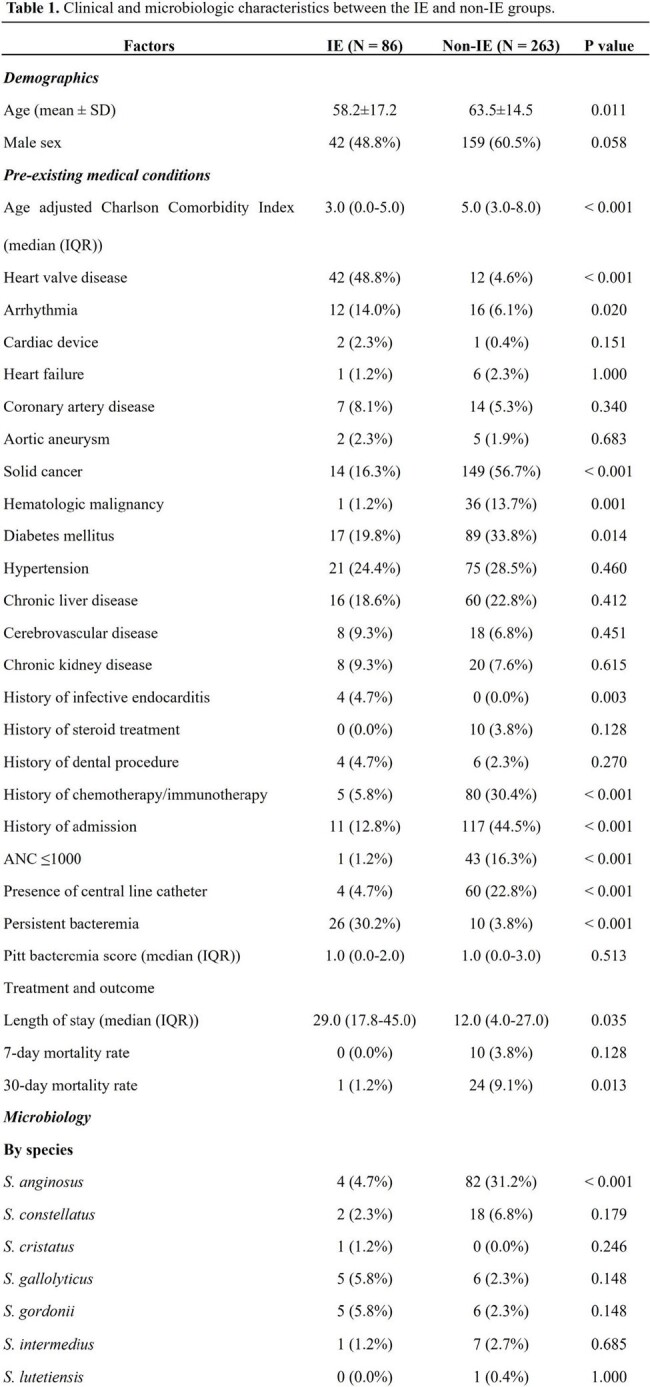

**Methods:**

This retrospective study conducted at Seoul National University Hospital from January 2013 to December 2022 involved patients with VGS and nutritionally variant streptococcal BSIs, excluding single positive blood cultures and polymicrobial BSI cases. Independent risk factors were identified by multivariate logistic regression and sensitivity analyses according to echocardiography results, VGS species, or the inclusion of possible IE cases.

Proportions of infective endocarditis by viridans group streptococci species.

**Figure:**
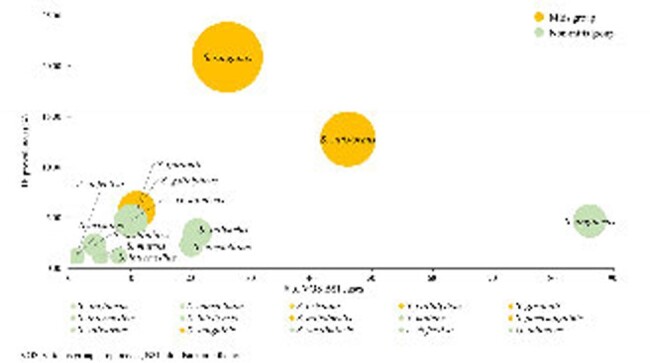

VGS species belonging to the mitis group are represented by the orange-coloured dots, and those in the non-mitis group are represented by the green-coloured dots. Circle size correlates with the number of IE cases attributed to each VGS species.

**Results:**

Of 845 VGS BSI cases, 349 were analyzed and 86 IE cases were identified (24.6%). In the multivariate analysis, heart valve disease (adjusted odds ratio [aOR], 14.14, 95% confidence interval [CI], 6.14–32.58; P < 0.001), persistent bacteremia (aOR, 5.12, 95% CI, 2.03–12.94; P = 0.001), age (per year, aOR, 0.98; 95% CI, 0.96–1.00; P = 0.015), solid cancer (aOR, 0.26, 95% CI, 0.13–0.53; P < 0.001), and hematologic malignancy (aOR, 0.04; 95% CI, 0.01–0.41; P = 0.006) were independently associated with IE. Sensitivity analyses yielded consistent results; also, infection by a member of the mitis group was independent risk factor for IE (aOR, 6.50; 95% CI, 2.87–14.68, P < 0.001).

**Figure:**
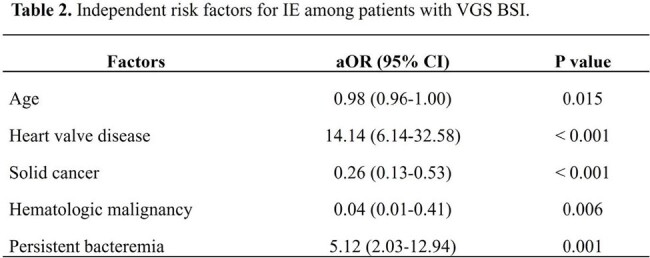

**Conclusion:**

Younger age, heart valve disease, persistent bacteremia, absence of underlying hematologic malignancy or solid cancer, and BSI by a member of the mitis group were the independent risk factors for IE in VGS BSI patients. In conclusion, regular echocardiographic evaluation could be prudently considered based on these clinicomicrobiological risk factors.

**Figure:**
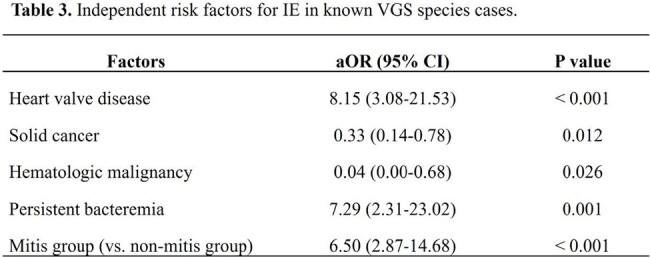

**Disclosures:**

**All Authors**: No reported disclosures

